# Solvates of New Arylpiperazine Salicylamide Derivative-a Multi-Technique Approach to the Description of 5 HTR Ligand Structure and Interactions

**DOI:** 10.3390/ijms22094992

**Published:** 2021-05-08

**Authors:** Edyta Pindelska, Anna Marczewska-Rak, Jolanta Jaśkowska, Izabela D. Madura

**Affiliations:** 1 Department of Analytical Chemistry and Biomaterials, Faculty of Pharmacy, Medical University of Warsaw, Banacha 1, 02-093 Warsaw, Poland; 2 Scientific Circle “Spektrum” at Department of Analytical Chemistry and Biomaterials, Faculty of Pharmacy, Medical University of Warsaw, Banacha 1, 02-093 Warsaw, Poland; ania.14.08@gmail.com; 3 Department of Organic Chemistry and Technology, Faculty of Chemical and Engineering and Technology, Cracow University of Technology, 24 Warszawska Street, 31-155 Cracow, Poland; jolanta.jaskowska@pk.edu.pl; 4 Faculty of Chemistry, Warsaw University of Technology, Noakowskiego 3, 00-664 Warsaw, Poland

**Keywords:** salicylamide, arylpiperazine, LCAPs, 5-HT1A and 5-HT7 receptors, solvates, X-ray crystallography, solid-state NMR, periodic DFT calculations, intermolecular interactions, hydrogen bonding

## Abstract

A new ligand for 5-HT1A and 5-HT7 receptors, an arylpiperazine salicylamide derivative with an inflexible spacer, is investigated to identify preferred fragments capable of creating essential intermolecular interactions in different solvates. To fully identify and characterize the obtained crystalline materials, various methods including powder and single-crystal X-ray diffraction, solid-state NMR, and thermal analysis were employed, supplemented by periodic ab initio calculations. The molecular conformation in different solvates, types, and hierarchy of intermolecular interactions as well as the crystal packing were investigated to provide data for future research focused on studying protein–ligand interactions. Based on various methods of crystal structure analysis, including the interaction energy calculation and programs using an artificial neural network, a salicylamide fragment was found to be crucial for intermolecular contacts, mostly of dispersion and electrostatic character. A supramolecular 2D kite-type layer of {4,4} topology was found to form in crystals. The closed voids between layers contain disordered solvents, very weakly interacting with the molecule and the layer. It has been postulated that the separation of the layers might be influenced by an increase in temperature or the size of the solvent; hence, only methanol and ethanol hemi-solvates could be obtained from a series of various alcohols.

## 1. Introduction

Synthesis of a new active pharmaceutical ingredient (API) is very often a challenge and many attempts are made to obtain pure, well-crystallized material with a defined structure. During the crystallization process, both the choice of the solvent and the process itself can affect the final product. Phenomena such as polymorphism or co-crystallization of the solvent molecules always have to be considered. Obtaining new polymorphic forms or solvates is sometimes the desired effect, especially from an economic point of view related to the possibility of securing new patents, but new forms must be carefully tested for pharmaceutical properties, such as, e.g., solubility, hygroscopicity, or thermal stability [1,2]. Concerning the solvated solids for pharmaceutical applications, one must remember that only a limited number of solvents are officially approved as safe [3]. On the other hand, the presence of a solvent molecule may sometimes influence a molecular structure of an API. As a rule, this does not much affect the therapeutic properties of the compound itself, but for research related to the modeling of ligand–protein interactions or chemical modifications of a given drug, the obtaining and characterizing of new solvates or polymorphs is extremely valuable. The broader the landscape of API conformations, the more precise are further studies using modern algorithms based on big data; for example, for ligand–protein interaction predictions [4], protein crystal structure refinement [5], or drug discovery [6].

**Scheme 1:**
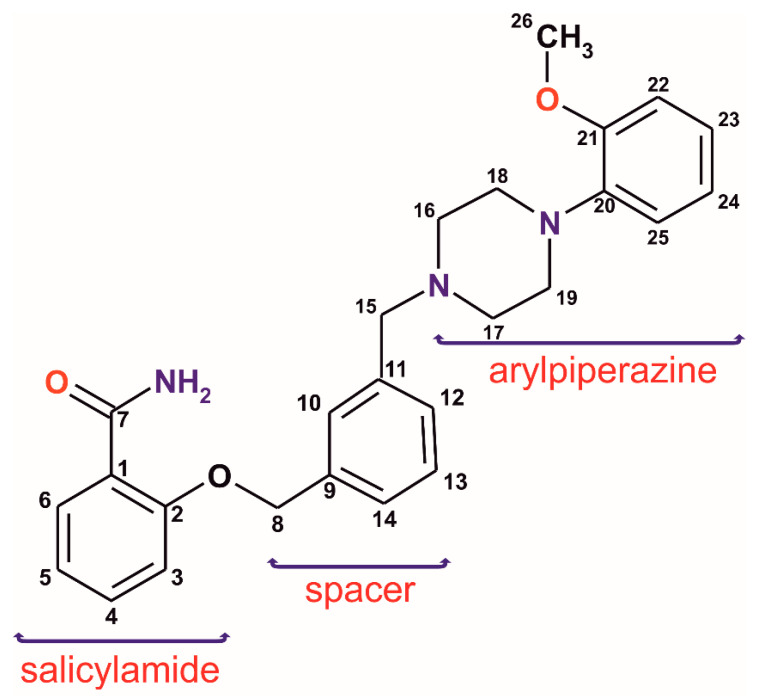
Analyzed salicylamide long-chain arylpiperazine derivative. The numbering scheme used in ssNMR and scXRD analyses.

Salicylamide derivatives are mainly known in medicine as analgesics but have recently also become the subject of research in the context of new serotonin receptor (5-HTR) ligands from the long-chain arylpiperazine group (LCAPs) [7,8]. In the case of derivatives containing arylpiperazine fragment, the structure–activity relationship studies focus on three main structural parts: the aryl group at the nitrogen atom of the piperazine ring, an aliphatic chain at the second N atom of the piperazine (a “spacer”), and a terminal fragment which has an amide or imide moiety (Scheme 1). In our previous publication concerning LCAPs [9], we observed a minor role of the spacer (the alkyl chain, (CH_2_)_n_–, with n = 4 ÷ 6) in the most important intermolecular interactions. Furthermore, the longer spacer tends to the positional disorder. Interestingly, not only the length of the chain but also a stiffening of the spacer, for example in the form of a xylene fragment, may influence the ligand activity. Studies on the LCAPs group containing a phthalimide fragment showed that the introduction of the *m*-xylene system results in a decrease in binding to the 5-HT_1A_ and 5-HT_7_ receptor [10]; however, in the case of analogous derivatives of salicylamide, a twofold increase in binding to the 5-HT_7_ receptor was detected [11]. Unfortunately, there are no crystal structures of these two receptors determined to be up-to-date; therefore, only pharmacophore models can be used for structure-based ligand–receptor analysis. Based on pharmacophore models for 5-HT_1A_ [12,13] and 5-HT_7_ [14,15] receptors, the effective arylpiperazine ligands have to contain a protonated nitrogen atom able to form charge-assisted hydrogen bonds, two or three hydrophobic/aromatic regions linking and two hydrogen bond acceptors. These molecular fragments of the arylpiperazine ligands are therefore expected to form the strongest interactions, also in the crystal. It is important to note that the ligand rarely binds to a receptor with strong covalent bonds but rather with weak reversible noncovalent interactions, so knowledge about possible interactions which may be formed by the new ligand seems to be very useful for the prospect of designing effective drugs. It is worth mentioning that the protonation seems to have only a slight influence on geometrical parameters of the arylpiperazine derivatives [9]. The cationic ligands are present in salts, which are a popular form for weakly soluble drug bioavailability improvement. Moreover, the ligands easily protonate in the stomach when pure compounds (bases) or their solvates are orally administrated.

In the work presented herein, we investigate the structural aspects of a new potential ligand with an inflexible spacer, namely 2-({3-[(4-(2-methoxyphenyl)piperazin-1-yl)methyl]benzyl}oxy)benzamide (**1**), currently studied in terms of enhanced receptor affinity. To fully identify and characterize the ligand (**1**), various methods including single-crystal X-ray diffraction (scXRD), powder X-ray diffraction (PXRD), solid-state nuclear magnetic resonance (ssNMR), and thermal analysis have been employed. The experimental studies have been additionally supplemented with ab initio calculations giving an insight into new solvates as well as ^13^C and ^15^N NMR shielding constants. Noteworthy is the ability of such a multitechnique approach to describe the molecular and crystal structures, particularly in the case of problematic crystals in scXRD experiments [16,17]. The ssNMR technique provides information about the near vicinity of the studied atoms in a complementary way to the data obtained from diffraction techniques. Hence, reliable information on hydrogen bonds formed in molecular crystals can be obtained, mainly due to the possibility of observing changes in nuclear shielding and chemical shifts of functional groups directly involved in these bonds. Even very weak interactions, particularly those with C-H donors, sometimes overlooked in the presence of stronger contacts, might be detected [18,19]. Herein, we have focused on (i) the ligand conformation in different solvates, (ii) types and hierarchy of the intermolecular interactions and their affinity to binding hydrophobic and/or hydrophilic molecular fragments, (iii) the crystal packing.

## 2. Results

Compound **1** is sparingly soluble in water; hence, a series of organic solvents was investigated to obtain crystalline material for structural studies. Methanol (MeOH) and ethanol (EtOH) water solutions were used first, followed by propanol, isopropanol, and *n*-butanol. The higher alcohols resulted in oils, while the methanol and ethanol deposits were white solids in the form of a fine crystalline precipitate.

**Figure 1:**
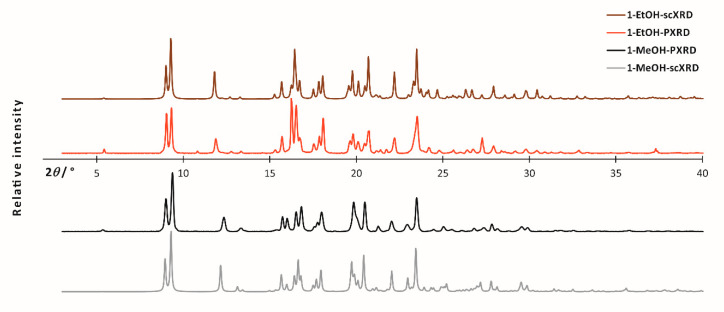
Experimental bulk powder X-ray diffraction patterns (black and red) and simulated powder diffraction patterns obtained from scXRD data (gray and brown) for **1-MeOH** and **1-EtOH**, respectively.

In the case of powdered/polycrystalline samples, PXRD and ssNMR techniques were the best to characterize the material. The powder X-ray diffraction measurements revealed the samples to be crystalline, with a similar pattern, however with discernible differences (Figure 1).

**Figure 2:**
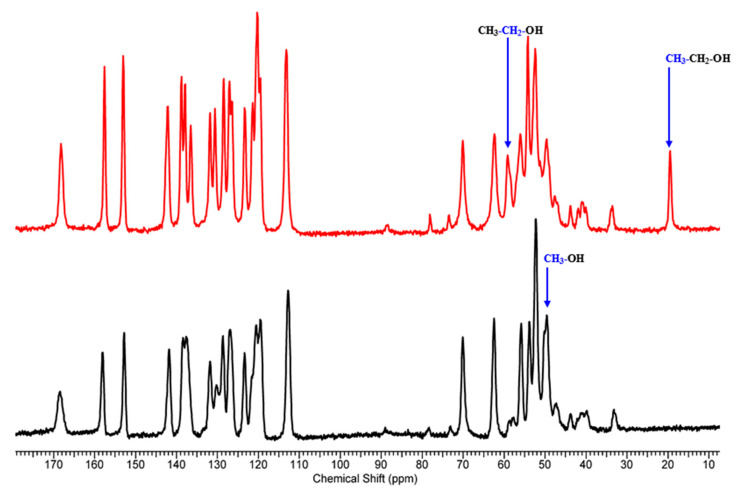
^13^C CP/MAS NMR spectra of **1-MeOH** (black) and **1-EtOH** (red) acquired with the contact time of 4 ms.

Next, we performed ssNMR measurements to confirm different solvates and elucidate the differences between them. To do so, one needs good quality ^13^C spectra with properly assigned peaks. The peak assignments (Table S1) were conducted based on the interpretation of the ^13^C NMR and ^1^H NMR solution spectra of **1** (Figures S1 and S2). The interpretation of the ^13^C CP/MAS NMR spectra for **1-MeOH** and **1-EtOH** (Figure 2) was conducted by reference to the solution chemical shifts and by considering the dipolar dephased spectra. Dipolar dephasing experiments are usually very helpful in assigning the ^13^C CP/MAS NMR spectra of solid APIs as they display signals arising from ^13^C nuclei undergoing weak dipolar interactions with protons: from quaternary carbons (no adjacent protons) and methyl carbons (group rotation). The dipolar-dephased spectra of **1-MeOH** and **1-EtOH** are presented in Figures S3 and S4. The obtained results indicated that two different solvates were formed. Interestingly, the ^15^N chemical shifts are similar in both spectra (**1-MeOH**: −269.86 ppm, −324.44 ppm, and −330.97; **1-EtOH**: −268.75 ppm, −324.96 ppm, −331.56 ppm). This may indicate that the solvents occupy the same positions in the crystal structure.

**Figure 3:**
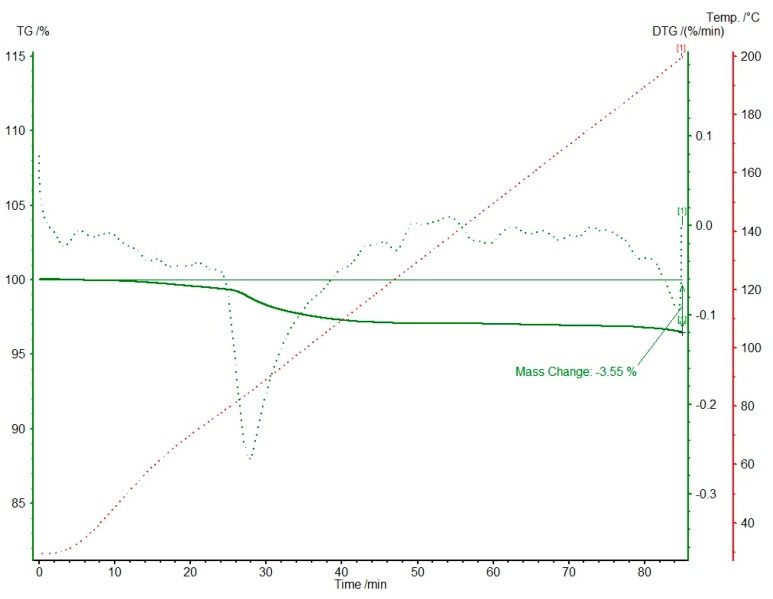
Thermogravimetric studies for **1-MeOH** (TG and DTG in %). The mass loss of 3.55% corresponds to the weight of 0.5 methanol molecule. Green solid and dotted lines correspond to TG and DTG, respectively. The red dotted line shows temperature changes in time.

Knowing that two different solvates, **1-MeOH** and **1-EtOH**, are formed, we focused on obtaining high-quality single crystals. In both cases, we obtained good enough crystals to perform scXRD experiments (Table S2). Nevertheless, in both solvates, we were unable to precisely refine the solvent positions. Not only does it reside near the symmetry center, but it is severely disordered. For **1-MeOH** we repeated the scXRD experiment at a lower temperature (120 K) to elucidate the nature of the disorder (**1-MeOH-low**). The results indicated the static disorder, without any better refining of the solvent molecule atom positions. The best as well as most reasonable refinement was obtained for both solvates with: (i) the fixed partial occupancies of the solvent atoms to 0.5; (ii) atoms refined only isotropically with *U*_eq_ fixed to 0.15 and 0.10 for **1-MeOH** and **1-EtOH**, respectively; (iii) in the case of ethanol molecule, restrained idealized geometry was applied [20]. We postulated half of the solvent molecule per one molecule of **1**. The SQUEEZE procedure [21] implemented in Olex-2 suite [22] substantiated that the final residual density corresponds to the number of electrons in 0.5 methanol or ethanol moiety in **1-MeOH** and **1-EtOH**, respectively (Table 1). The final proof of the half solvent molecule per drug molecule was confirmed by thermogravimetric studies. The data for **1-MeOH** are shown in Figure 3. The mass loss of 3.55% corresponds to half of the methanol molecule and occurs in c.a. 30 min at a temperature less than 85 °C. No other mass loss was detected up to 200 °C. Additionally, on the DSC curve (Figure S5), an endothermic effect of approximately 80 kJ/mol has been detected at 88 °C and might be connected with the solvent loss.

Given the ssNMR data and the fairly well-refined structures, we performed periodic DFT calculations. For a detailed comparison, two sets of calculations were carried out: (i) with optimization of hydrogen and heavy atom positions and (ii) with the addition of unit cell optimization. The advantage of the calculation is the possibility to model other, similar structures. Hence, except for the analyzed solvates (**calc 1-MeOH** and **calc 1-EtOH**), we also calculated the probable crystal structures of pure compound **1** (**calc 1**) as well as a monohydrate (**calc 1-H_2_O**). In Table 1, the unit cell and packing parameters are collected.

**Figure 4:**
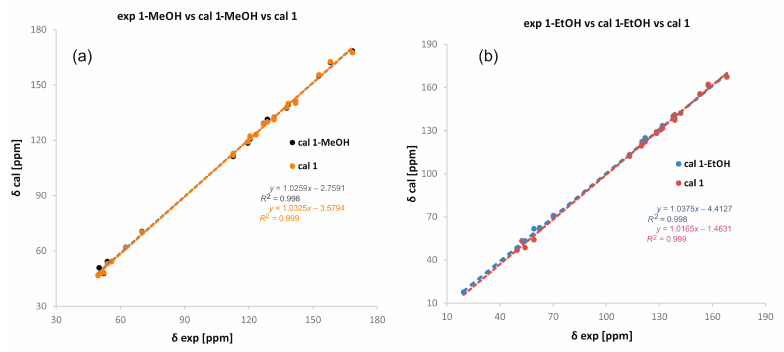
Correlation between experimental and theoretical chemical shifts of carbon-13 in **1-MeOH** (**a**) and **1-EtOH** (**b**). Theoretical δ values have been calculated in CASTEP [26] for structures with optimized all atom positions and the lattice parameters fixed to experimental values. Two sets of structures have been considered: with one solvent per molecule of **1** and without solvent.

However, CASTEP [24,25] procedures do not include short-range ordering and ignore partial occupancies of any fragments. Thus, the local distortions around the atoms are not taken into account and therefore such calculations do not reproduce the finer details of the disordered structures very accurately. To reliably compare the experimental NMR data with the calculated ones, we performed calculations for monosolvate structures (i.e., one solvent per molecule) and “pure compounds” (no solvent and unit cell parameters not optimized). For both solvates, we obtained perfect correlations regardless of whether we took the data for the monosolvate or the pure compound (Figure 4). This may indicate that the solvent weakly interacts with the molecules and the supramolecular framework formed by molecules of **1** remains intact despite the presence or absence of the solvent.

**Figure 5:**
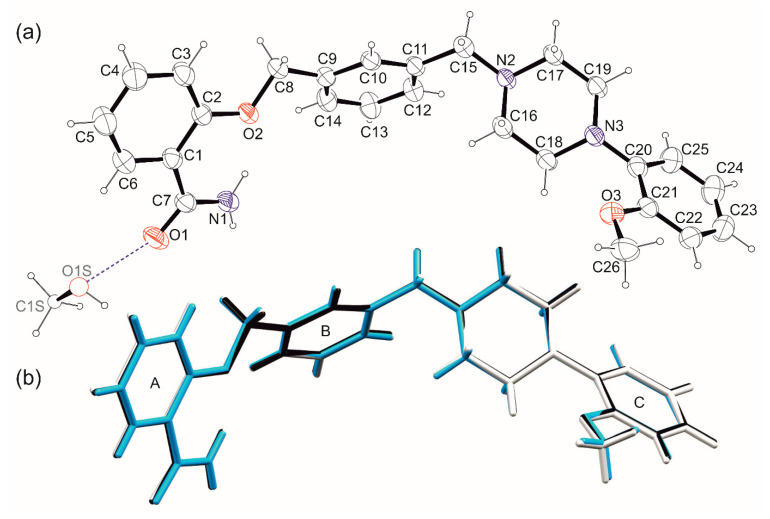
(**a**) Ortep [27] drawings of the **1-MeOH** asymmetric unit with the atom numbering scheme. Thermal ellipsoids are presented at 30% probability. Methanol molecule with partial occupancy restrained to 0.5 is depicted with spheres of arbitrary radius. (**b**) Overlay of molecules of **1** from **1-MeOH** (black), **1-MeOH-low** (white), and **1-EtOH** (turquoise) crystals. Aromatic rings are numbered with capital letters A–C.

The comparison of the results of scXRD studies for both solvates revealed that the geometry of molecule of **1** remains unchanged regardless of the solvent type or lowering the temperature of the measurement (Figure 5b). The first part of the molecule (Scheme 1, Figure 5) consists of the aromatic amide ring (A) coplanar with O2–C8 fragment. The deviation from the l.s. plane defined by C1–C8, O1–O2 and N1 atoms does not exceed 0.050(2) Å in all analyzed structures. The second aromatic ring (B) constitutes a spacer with two CH_2_ fragments in *meta* position. The spacer is essentially flat with deviation from the planarity not bigger than 0.036(3) Å. The salicylamide fragment and spacer planes are oriented to each other at an angle of ca 40°. The last part of the molecule contains a saturated piperazine ring in a chair conformation and the third aromatic ring (C) with methoxy substituent in *ortho* position concerning the N3-C20 bond. The ring C forms dihedral angles of ~75 and ~65° with the salicylamide and spacer planes, respectively.

Although the data from the XRD and ssNMR experiments as well as the DFT calculations point out a very similar spatial structure of different solvates, even small differences might be of importance in future investigations concerning a new derivative design or elucidation of the ligand–protein interactions. Therefore, we thoroughly investigated the interactions in which the molecule of compound **1** is involved in the crystals characterized by the scXRD method and those fully optimized. First of all, we detected the main motifs formed via various types of weak interactions and described them with the graph sets proposed by Etter [28]. Due to the observed disorder, the interactions of solvates with molecules of **1** in both crystals were not analyzed. However, we found out that in calculated structures, methanol and ethanol molecules are weakly bound via O-H…O hydrogen bonds to the amide oxygen atom (Table S3). The estimated intermolecular potentials [29,30] of the interactions between **1** and the solvent are −7.34 kJ/mol and −12.1 kJ/mol for **calc 1-MeOH** and **calc 1-EtOH**, respectively. In the hydrate, a water molecule is more strongly bound to the molecule of **1** with an energy of −21.4 kJ/mol. No meaningful interactions between solvent molecules were detected.

**Figure 6:**
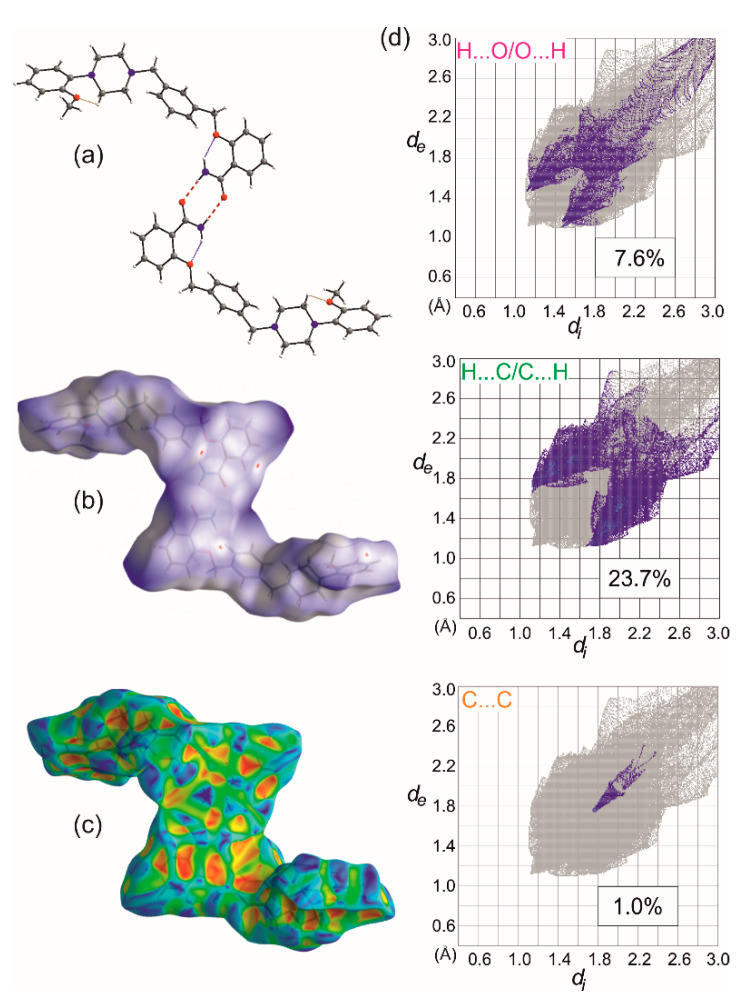
Basic dimeric *R*^2^_2_(8) motif formed by N-H…O bonds (red dashed lines) and intramolecular interactions leading to *S*(6) motifs (dotted lines). (**a**). Hirshfeld surface calculated for the dimer with depicted *d*_norm_ (**b**), and shape index properties (**c**), and selected decomposed fingerprint plots showing characteristic features of C-H…O, C-H… π and π … π interactions (**d**).

In all structures, the basic structural motif is a centrosymmetric *R*^2^_2_(8) dimer formed by the amide group (N1-H1A…O1, Figure 6a, Table S3). Additionally, the second amide hydrogen atom (H1B) is engaged in the intramolecular hydrogen bond with O2 atom, resulting in an *S*(6) motif, which due to coplanarity with the aromatic ring A may be regarded as a resonant assisted H-bond [31]. In calculated structures, both N-H…O bonds are somewhat shorter, yet the angle remains generally the same (Table S3). In the molecule of **1**, weak C18-H18…O3 intramolecular hydrogen bonds between piperazine ring and methoxy substituent can be distinguished, also described with an *S*(6) graph (Figure 6a).

**Figure 7:**
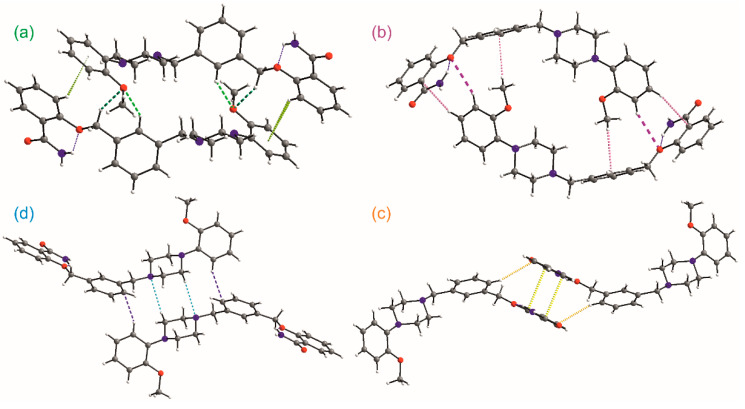
Main motifs formed by weak intermolecular interactions (**a**–**d**).

To decode weak interactions between the basic 0D supramolecular assembly, i.e., dimer with the weakly bounded solvent molecules, the Hirshfeld surface (HS) analysis [32] has been performed for the dimer (the experimental structures refined with the SQUEEZE procedure were taken). There are no meaningful differences in the HS analysis for experimental structures; hence, the data for **1-MeOH** are mostly presented. In Figure 6b, HS with depicted *d*_norm_ properties [33] is presented and the small red spots correspond to interactions between C-H donors from each of the aromatic rings and O2/O3 oxygen atoms acting as the acceptors. The found C-H…O motifs (Figure 7) are described with *R*^2^_2_(22) (light green, Figure 7a), *R*^2^_2_(26) (dark green, Figure 7a), and *R*^2^_2_(28) (dark pink, Figure 7b) graph descriptors. The HS mapped with the Shape Index [33] (Figure 6c) also discerns the importance of interactions with aromatic rings. The significance of these interactions has been confirmed by the Aromatics Analyzer, a module implemented in the Mercury 2020.2.0 program [23], which uses an artificial neural network to quantify the interactions between aromatic rings (the score is 0–3 for weak, 3–7 for moderate, and 7–10 for strong interactions, respectively). The results indicated two C-H… π interactions (with the ring slippage around 60°) to be strong ones, with the mean score of 9.2 and 7.4, respectively (Table S4). They are present in two dimeric motifs colored green and pink in Figure 7a,b, respectively. In these dimeric motifs, C-H…O interactions (these visible on HS, Figure 6b) and C-H… π contact between the methyl group and aromatic ring B strengthen the motifs. The Aromatic Analyzer calculations also pointed out the importance of face-to-face contact of antiparallel-oriented aromatic rings A (a yellow motif in Figure 7c). Interestingly, in all methanol solvate structures, this interaction is ranked as moderate with a score of 6.1–6.6, whereas in ethanol solvate and hydrate it is somewhat stronger, and in the calculated pure crystal (**calc 1**) and **calc 1-EtOH** it is classified as strong. Additionally, this stacking interaction is supported by C-H… π contact, ranked as moderate (the score 4.9–5.6), which is stronger when the π…π interaction gets weaker (Table S4). In Figure 6d, the HS surface is presented in the form of decomposed fingerprint plots [33] for O…H/H…O, C…H/H…C, and C…C contacting atoms, which correspond to C-H…π, C-H…O, and π…π weak interaction types. The shape of the highlighted fingerprint plots, as well as the percentage of the total HS, indicate that C-H…π interactions are the predominant contacts in the organization of basic dimers into a crystal.

**Figure 8:**
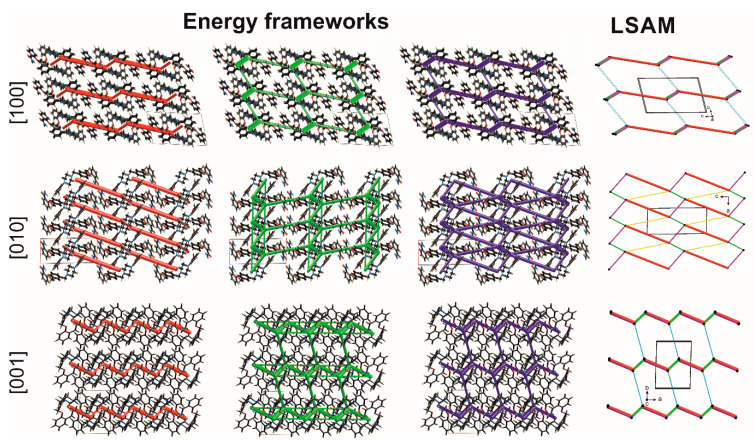
Energy frameworks in **1-MeOH**: electrostatic (red) and dispersion (green) contributions to the total energy (blue). Topology of the network (LSAM): black dot represents the center of gravity of molecule 1, lines in agreement with directions of intermolecular interactions (colors correspond to motifs in Figure 7). Views along unit cell basis vectors.

Taking into account that even weak intermolecular interactions are directional, one can analyze the topology of the supramolecular network where a molecule can be reduced to a node (vertex), usually being a center of gravity of the molecule. The links between nodes are therefore in agreement with the meaningful interactions’ directions. In the case of **1**, all three motifs (Figure 7a–c) and the basic dimer constitute a layer parallel to the (010) plane. It is a 4-connected uninodal 2D kite-type network described with the {4,4} Schläfli symbol or 4^4^ vertex configuration [34] (see the middle picture in the fourth column in Figure 8).

To more precisely discern the hierarchy of the synthons, we calculated at the DFT (B3LYP/6–31 G(d,p)) level of theory the energy of intermolecular interactions using the energy frameworks module [35] implemented in the Crystal Explorer program [36]. The total energy is given as the sum of the four energy components: electrostatic energy, polarization, dispersion, and exchange-repulsion (Table S5). The biggest electrostatic contribution (around −70 kJ/mol) is associated with the formation of the first level of the crystal structure, i.e., 0D dimer. However, a very strong repulsion causes these interactions to be the second in line (the total energy ca ‒60 kJ/mol) after the interaction in the green motif (note the strongest C-H…π hydrogen bond), where both electrostatic (~−30 kJ/mol) and dispersion (~−100 kJ/mol) components contribute to the total energy of −90 kJ/mol. The dispersion forces are dominant for C-H…O, C-H…π, and π…π interactions in pink and yellow motifs, both showing the total energy around −55 kJ/mol. Following the calculated energies (Table S5), the geometry of all motifs (Table S3), and results from the Aromatic Analyzer, the 2D layer can be regarded as a large synthon (Long-Range Synthon Aufbau Module, LSAM [37]), which provides information on symmetry, long-range order, and overall topology of the crystal. Projection along the [10] axis in Figure 8 allows the topology (LSAM) and the energy components of the layer to be traced. Important to note is that the shape of LSAM is consistent with the calculated energy frameworks. This means that the indicated intermolecular interactions are the most important in which the molecule of **1** may be involved, probably also in biological systems. The energy calculations also revealed that the interlayer interactions of mostly dispersion character have energy around −35 kJ/mol. They agree with weak C-H…N hydrogen bonds (the *R*^2^_2_(8) blue motif in Figure 7, Table S3) and are denoted as thin blue lines in the topology representation. Moreover, the quantified as moderate aromatic contacts occurring between LSAMs exhibit energy around 20 kJ/mol. Interestingly, for **calc 1-EtOH** the interlayer interactions are reduced to only one contact between aromatic rings with a score of 5.8, i.e., moderate.

**Figure 9:**
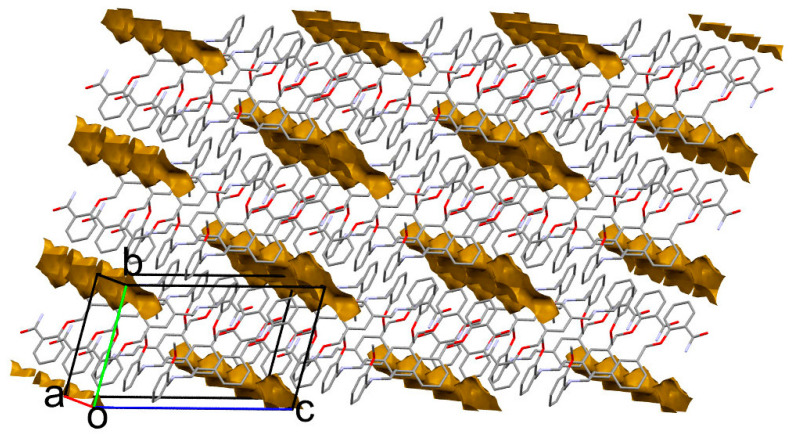
Packing of molecules in **1-MeOH** crystal. The closed voids (brown shapes) indicate the solvent accessible volume. Hydrogen atoms are omitted for clarity.

A closer inspection of the crystal packing disclosed the presence of closed voids accessible for a solvent (Table 1, Figure 9). In the **calc 1** structure, there is still some empty space between layers, capable of holding a very small solvent, for example, a water molecule; nevertheless, **1** is practically insoluble in water. It is worth noting that the presence of a solvent in the calculated solvates results in very close-packed structures with the highest packing coefficient [38] of 73.3 for **calc 1-MeOH**. On the contrary, artificial solvent removal leads to a packing index as low as 62.3 for **1-MeOH**. This may indicate that even though the solvents are weakly bound to the molecules, they are necessary to fill the space between the layers. Notably, the accessible solvent volume drops with temperature, so one might assume that during the heating process the LSAM separates and the solvent might be released. Moreover, a solvent as large as ethanol that fully fills the voids (**calc 1-EtOH**) may lead to the layers’ detachment and structure destabilization (Figure S6). This, in turn, might explain why attempts to crystallize **1** from higher alcohols resulted in oils.

**Figure 10:**
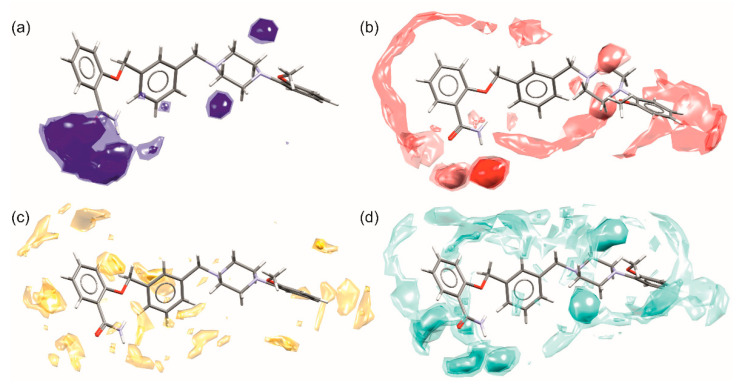
Full interaction maps of **1** showing the most probable contacts with (**a**) hydrogen bond donors, (**b**) hydrogen bond acceptors, (**c**) C-H donors and aromatic fragments, (**d**) water molecules. The darker color or small balls (hot spots) correspond to the most probable spatial placement of an important intermolecular interaction.

The scrutinized analysis of weak interactions in both solvates of **1** pointed out the most important contacts in which the particular fragment of the molecule is engaged. In Figure 10 the full interaction maps are presented [39]. They indicate the interaction preferences and correspond well to pharmacophore models for 5-HT_1a_ [12] and 5-HT_7_ [13] receptors. Important to note are the areas near piperazine nitrogen atoms, pointing out that this fragment can be either hydrogen bond acceptor or donor depending on protonation (Figure 10a,b,d). In all pharmacophore models as well as crystal structures of other 5HTR with piperazine ligands, the charge assisted hydrogen bonds between protonated N2 atom and carboxylate group from aspartate (Asp3.32) are regarded as crucial for ligand binding. The hot spots on both sides of the aromatic ring (Figure 10c) of ortho-methoxyphenyl substituent, as well as the ability of this fragment to act as a C-H donor to electron-rich groups (either aromatic fragments or carbonyl oxygen atoms), indicate the capability of this fragment to form C-H…π and π…π interactions. In pharmacophore models, this fragment binds to various amino acids, either hydrophobic phenylalanine (Phe 6.52, Phe 3.28) or hydrophilic tyrosine (Tyr 7.43). In some models, the methoxy substituent is regarded as forming a weak hydrogen bond with Ser 5.42. On the interaction maps, this fragment seems to remain inactive, in contrast to the interactions in the discussed crystals, where the OCH_3_ group is involved in weak C-H…O interactions (the green motif in Figure 7). The salicylamide is a good example of a molecular fragment capable of hydrogen bonding, and pharmacophore models show that it may interact with asparagine (Asn 7.39) in the 5-HT_1a_ receptor or threonine (Thr 5.43) and serine (Ser 5.42) in the 5-HT_7_ receptor. The maps also show that this fragment is very active in aromatic interactions: in crystals of **1** it is engaged in the strongest C-H…π interactions while in receptors it may interact with phenylalanine (Phe 3.28), valine (Val 3.33), or isoleucine (Ile 4.56). Noteworthy is a third aromatic/hydrophobic fragment, i.e., the spacer, which may also form both types of aromatic interactions.

## 3. Materials and Methods

### 3.1. Sample Preparation

Compound **1**(2-({3-[(4-(2-methoxyphenyl)piperazin-1-yl)methyl]benzyl}oxy)benzamide) was synthesized according to the methods described previously [40]. The structure in solution and purity of the sample were confirmed by ^13^C and ^1^H NMR spectroscopy (for details see Supporting Information). To obtain high-quality single crystals for X-ray diffraction analysis, the slow solvent evaporation method has been chosen. The solvents—methanol, ethanol, propanol, isopropanol, and *n*-butanol were purchased from Avantor Performance Materials Poland S.A. (Gliwice, Poland) and used without purification.

### 3.2. Solid-State Nuclear Magnetic Resonance (ssNMR)

^13^C and ^15^N CP/MAS NMR spectra were recorded at room temperature with a Bruker Avance 400 WB (B_0_ = 9.4 T, Bruker, Germany) using 100.61 and 40.50 MHz resonance frequencies, respectively. The NMR experiments were performed using cross-polarization (CP), high-power decoupling, and magic-angle spinning (MAS) using a Bruker 4.0 mm HX CP/MAS probe with zirconia rotors driven by dry air. The spin rates of 8 kHz and 5 kHz for ^13^C and ^15^N NMR experiments were used, respectively. The Hartmann–Hahn conditions for ^13^C and ^15^N were matched using glycine 15-N. The optimized recycle delays for **1-MeOH** and **1-EtOH** were 50 s, 40 s, respectively. The dipolar dephased experiments were carried out with dipolar filters to suppress the CP/MAS NMR signals from ^13^C nuclei strongly coupled to protons (CH and CH_2_ groups). Insertion of a 50 µs delay before the FID acquisition resulted in the selective dephasing of magnetizations from methine and methylene groups. The NMR spectra were processed with the ACD/SpecManager NMR program (version 10.0, Advanced Chemistry Development, Inc., Toronto, ON, Canada).

### 3.3. X-ray Diffraction

Single crystal X-ray diffraction (scXRD) data for **1-MeOH** and **1-EtOH** were collected on a Rigaku Oxford Diffraction Gemini A Ultra diffractometer (Rigaku Corporation, Tokyo, Japan) using mirror monochromated CuKα radiation (λ = 1.54184 Å) or graphite monochromated MoKα (λ = 0.71073 Å). Measurements for both samples were performed at room temperature and additionally at 120 K for **1-MeOH**. Cell refinement and data collection as well as data reduction were performed with CrysAlisPro software [41]. The empirical absorption corrections using spherical harmonics, implemented in the multiscan algorithm, were performed. The structure was solved with the SHELXD [42] structure solution program using Dual Space and refined with the SHELXL [43] refinement package using least squares minimization, both implemented in the OLEX2 suite [22]. All non-hydrogen atoms were refined anisotropically. H-atoms on C atoms were added to the structure model at geometrically idealized positions and refined as riding atoms, with *U*_iso_(H) = 1.2 × *U*_eq_(CH) and 1.5 × *U*_eq_ (CH_3_). H-atoms of amino groups were located from a Fourier map and refined with restraints on the N-H distances of 0.88(1) Å. Molecular diagrams were generated using ORTEP-3 for Windows [27] (Figure 5a), Diamond [44] (Figure 7, LSAMs in Figure 8), and Mercury 2020.2.0 [23] (Figure 5b and Figure 9, and Figure S6) programs, whereas geometrical parameters were calculated using the Platon package [45,46]. The crystal data and structure refinement parameters are given in Table S2.

Powder X-ray diffraction (PXRD) samples were recorded at room temperature on Bruker Advance D8 diffractometer (Bruker, Billerica, MA, USA) equipped with an LYNXEYE position-sensitive detector using Cu Kα radiation (λ = 1.5418 Å). The data were collected in Bragg–Brentano (θ/θ) horizontal geometry (flat reflection mode) between 3 and 45°(2θ) in a continuous scan using 0.03° steps and 384 s per step. The diffractometer incident beam path was equipped with a 2.5° Soller slit and a 1.14° fixed divergence slit, while the diffracted beam path was equipped with a programmable anti-scatter slit (fixed at 2.20°), a Ni β-filter and a 2.5° Soller slit.

### 3.4. Thermal Analysis

Differential scanning calorimetry (DSC) studies were performed using a TA Instruments Q200DSC apparatus (New Castle, DE, USA) in nitrogen flow. The heating rate was equal to 3 °C/min, the sample mass was about 4–5 mg, and the temperature range was 20–1380 °C. The samples were sealed in an aluminum standard sample pan. Indium (m.p., T = 429.76 K) was used as the standard for the DSC calibration. DSC results are available in Supplementary Material (Figure S5).

Thermogravimetric studies (TGA) were performed using Netzsch STA449C thermobalance. The samples were heated gradually from room temperature up to 200 °C at the constant rate of 2 °C/min.

### 3.5. GIPAW DFT Calculations

The density functional theory (DFT) calculations of geometry optimization at 0 K and NMR shielding constants were carried out with the CASTEP program [47] implemented in the Materials Studio 2017 software (Accelrys Software Inc., San Diego, CA, USA) [24] using plane-wave pseudopotential formalism and the PBE exchange-correlation functional [48], defined within the GGA with Tkatchenko–Scheffler method for the dispersion (DFT-TS) correction [49]. The crystal structure of **1-MeOH** and **1-EtOH** determined by scXRD was used as a starting point for the geometry optimization. In the calculations, the positions of all atoms were optimized, while the cell parameters were either fixed to their experimental values or refined. All the calculations were performed with ultrasoft pseudopotentials calculated on the fly; the quality of calculations was set to fine as implemented in the CASTEP standards. CASTEP default values for the geometry convergence criteria were used. The kinetic energy cutoff for the plane waves was set to 550 eV. Brillouin zone integration was performed using a discrete 2 × 2 × 1 for Monkhorst–Pack k-point sampling for a primitive cell. The computations of shielding tensors were performed using the gauge including projector augmented wave (GIPAW) method of Pickard and Mauri [50]. To compare the theoretical and experimental data, the calculated chemical shielding constants (σiso) were converted to chemical shifts (δiso), using the following equation: δiso = (σGly + δGly) − σiso, where σGly and δGly stand for the shielding constant and the experimental chemical shift, respectively, of the glycine carbonyl carbon atom (176.5 ppm).

### 3.6. Hirshfeld Surface Calculations

The molecular Hirshfeld surfaces (HS) [32] presented in a form of *d*_norm_, shape index, and 2D-fingerprint plots [33] were calculated from crystal structure coordinates using the CrystalExplorer 17.5 program. [36] The surfaces were constructed based on the electron distribution calculated as a sum of spherical atom electron densities. For each point on the Hirshfeld surface, *d*_e_ (the distance from the surface to the nearest atom external to the surface) and *d*_i_ (the distance from the surface to the nearest atom internal to the surface) parameters were determined. In Figure 6a, the HS are mapped with *d*_norm_ (distances normalized with vdW radii for a given atoms pair) over the range of −0.5 to 1.5 with the red–white–blue coloring scheme. The 2D-fingerprint plots (Figure 6d) were derived from the HS by plotting the fraction of points on the surface as a function of the pair (*d*_i_, *d*_e_). The range of fractions spanning 0.05% of surface areas was used. The energies of intermolecular contacts for the 3D energy framework construction were calculated based on the single-point molecular wave function at B3LYP/6–31 G(d,p) level of theory within a cluster of a radius of 3.8 Ǻ. In Figure 8, cylinders were adjusted to the same scale factor of 60 with the energy cut-off value of 30 kJ/mol.

## 4. Conclusions

The conducted research shows how important in the analysis and description of the API crystal structure with disordered solvent is the use of complementary methods, both experimental and theoretical. The PXRD studies of **1** obtained from different solvents allowed us to discern subtle differences in the diffractograms while ssNMR experiments confirmed the solvent type. Single-crystal X-ray diffraction experiments revealed a severe solvent disorder and without the data from powder analyses, we would not have been able to unequivocally define both the type and amount of the solvent. The refinement with the SQUEEZE procedure as well as thermal analysis enabled us to postulate half of the solvent molecule in both solvates. Moreover, the scXRD measurement with temperature reduction allowed us to conclude that there is a static disorder present in the analyzed crystals. The observed disorder excluded analysis of the interactions of solvates with molecules of **1**; however, the rough estimation of these interactions could be conducted based on data from periodic DFT calculations. The correctness of these calculations was confirmed by the comparison of chemical shielding constants in ssNMR spectra and those obtained from GIPAW calculations.

Taking into account the promising activity of **1** towards binding to the 5-HT_1a_ and 5-HT_7_ receptor, we thoroughly investigated the interactions in which the ligand is involved in the crystals characterized by the scXRD method and those fully optimized. We focused on establishing a hierarchy of interactions in which the molecule is involved. Such an approach may help in the future interpretation of the results of ligand–receptor binding site analysis and targeted drug modifications. First of all, we found out that regardless of solvent or its absence, the molecular conformation of **1** remains unchanged and only small differences could be detected in the supramolecular framework. Using various methods of crystal structure analysis, from a meticulous analysis of geometry to the calculation of the interaction energy at the level of DFT theory and programs using artificial neural network methods, we have found that molecule (the salicylamide fragment) is engaged in the strongest interactions. Important to note is the role of the salicylamide aromatic ring which is likely to form very strong interactions with predominant dispersion character, overcoming even the hydrogen bonds formed by the amide group, where relatively strong repulsion may cancel the electrostatic component. In crystals, these interactions are present in the four most important dimeric motifs, which all constitute a Long-Range Synthon Aufbau Module in the form of a layer perpendicular to [10] crystallographic direction. The *m*-xylene spacer tends to enforce the interactions of the salicylamide fragment while the flexible arylpiperazine may adjust to the environment. In crystals, the arylpiperazine is involved in the interlayer interactions and our analysis revealed that, depending on a solvent, these interactions may be broken, probably preventing the crystal formation in the case of bigger solvents. However, in most pharmacophore models, the protonated aryl piperazine forms charge-assisted hydrogen bonds with the 5-HT receptors while other fragments have to be involved either in aromatic interactions or are the acceptors of hydrogen bonds. On full interaction maps of **1**, it is shown that these requirements are fulfilled. Hence, taking into account the model proposed by Kołaczkowski et al [14], the molecule of **1** in the conformation found in solvates seems to be a good candidate to obey the general affinity hypothesis for interactions with the 5-HT7 receptor, as well as due to additional fragment enhancing C-H…π interactions, it may also be an effective ligand for the 5-HT1a receptor [51].

## Figures and Tables

**Table 1:** Unit cell and packing parameters for experimental and calculated structures.

	1-MeOH	1-MeOH Low	1-EtOH	Calc 1-MeOH *	Calc 1-EtOH *	Calc 1-H_2_O *	Calc 1
*a*/Å	7.2792(4)	7.2165(4)	7.5240(3)	7.1704	7.3751	7.1916	7.1932
*b*/Å	10.1555(5)	9.9585(6)	10.1584(4)	10.1861	10.7564	9.7899	9.9247
*c*/Å	17.1001(7)	17.030(1)	16.9167(6)	16.9461	16.9272	17.0122	16.9496
*α*/°	76.527(4)	76.185(6)	75.404(3)	74.8319	69.7323	74.9742	75.1011
*β*/°	87.792(4)	87.712(5)	85.663(3)	88.3336	84.9575	87.4625	87.5922
*γ*/°	87.810(4)	87.901(5)	85.721(4)	89.8875	84.3890	88.7825	87.8934
V/Å^3^	1227.9(1)	1187.1(1)	1245.59(8)	1194.1	1251.5	1155.6	1167.9
Solvent accessible voids/Å^3^ **	34.5	25.8	42.1	24.5	43.1	17.4	19.7
Voids per unit cell /%	2.8	2.2	3.4	2.1	3.4	1.5	1.7
No. of electrons per void	19.3	16.9	29.0	–	–	–	–
Packing index	62.3	65.1	61.4	73.3	72.5	72.5	68.7

[*] Calculated structures with all atoms and unit cell parameters optimized. One molecule of solvent per molecule of **1** was considered. [**] For experimental structures refined with the SQUEEZE procedure, solvent-accessible voids were calculated using Mercury 2020.2.0 program [23]. The volume occupied by a solvent molecule in calculated solvates was calculated with the aid of Solvent Analyzer implemented in Mercury. In both cases, the probe radius was 1 Å and the approximate grid spacing was 0.5 Å.

## Supplementary Materials

The following are available online at https://www.mdpi.com/article/10.3390/ijms22094992/s1. PDF file: Table S1. ^13^C NMR Chemical Shielding Constants (ppm) in experimental 1-MeOH and 1-EtOH (Exp), Compared with those in calculated with and without solvent (Cal); Figure S1. The ^13^C NMR spectrum (in CDCl_3_ to 300 MHz) of 1; Figure S2. The ^1^H NMR spectrum (in CDCl_3_ to 300 MHz) of 1; Figure S3. ^13^C CP/MAS NMR spectra of the 1-MeOH: standard (bottom) and dipolar-dephased (top). Recorded with a contact time of 4 ms. The dipolar filter was set to 50 μs.; Figure S4. ^13^C CP/MAS NMR spectra of the 1-EtOH: standard (bottom) and dipolar-dephased (top). Recorded with a contact time of 4 ms. The dipolar filter was set to 50 μs; Table S2. Crystal data and structure refinement; Table S3. Hydrogen bonds and stacking interactions geometry (in Å and °) in experimental and calculated structures; Table S4. Aromatic analyzer score values for hierarchization of interactions between aromatic rings; Figure S5. DSC curve for 1-MeOH; Table S5. Interaction Energies (kJ/mol) calculated in Crystal Explorer program at B3LYP/6-31G(d,p) level of theory; Figure S6. The crystal packing of: (a) Calc 1-H2O, (b) Calc 1-MeOH, (c) Calc 1-EtOH. ZIP file contains data for calculated structures calc 1, calc 1-MeOH, calc 1-EtOH, and calc 1-H2O. CCDC 2074714-2074719 contains the supplementary crystallographic data for this paper. The data can be obtained free of charge from The Cambridge Crystallographic Data Centre via www.ccdc.cam.ac.uk/structures, accessed on 7 May 2021.

## Sample Availability

Samples of compounds **1-MeOH** and **1-EtOH** are available from the authors.
