# Supplementary material for: Solvates of New Arylpiperazine Salicylamide Derivative-a Multi-Technique Approach to the Description of 5 HTR Ligand Structure and Interactions
Source: Int J Mol Sci. 2021 May 8;22(9):4992. doi: 10.3390/ijms22094992 (PMC8125853; doi:10.3390/ijms22094992)
Supplement: Supplementary file 1 [file ijms-22-04992-s001.zip › Suplementary Materials.pdf]

International Journal of Molecular Sciences

Solvates of new arylpiperazine salicylamide derivative - a multi-technique approach to the description of 5-HTR ligand structure and interactions

Edyta Pindelska[1*], Anna Marczewska-Rak[2], Jolanta Jaśkowska[3], Izabela D. Madura[4*]

[1]Department of Analytical Chemistry and Biomaterials, Faculty of Pharmacy, Medical University of Warsaw, Banacha 1, 02-093 Warsaw, Poland

[2] Scientific Circle "Spektrum" at Department of Analytical Chemistry and Biomaterials, Faculty of Pharmacy, Medical University of Warsaw, Banacha 1, 02-093 Warsaw, Poland

[3]Department of Organic Chemistry and Technology, Faculty of Chemical and Engineering and Technology, Cracow University of Technology, 24 Warszawska Street, 31-155 Cracow, Poland

[4]Faculty of Chemistry, Warsaw University of Technology, Noakowskiego 3, 00-664 Warsaw, Poland

*Corresponding authors: Tel./fax: +48 (22) 57 20 784; +48(22) 2347272

E-mail address: edyta.pindelska@wum.edu.pl; izabela.madura@pw.edu.pl

Contents:

Page 3: **Table S1.** ^{13}C NMR Chemical Shielding Constants (ppm) in experimental **1-MeOH** and **1-EtOH** (Exp), Compared with those in calculated with and without solvent (Cal)

Page 4: **Figure S1.** The ^{13}C NMR spectrum (in $CDCl_3$ to 300 MHz) of **1**.

Page 4: **Figure S2.** The ^{1}H NMR spectrum (in $CDCl_3$ to 300 MHz) of **1**.

Page 5: **Figure S3.** ^{13}C CP/MAS NMR spectra of the **1-MeOH**: standard (bottom) and dipolar-dephased (top). Recorded with a contact time of 4 ms. The dipolar filter was set to 50 μs.

Page 5: **Figure S4.** ^{13}C CP/MAS NMR spectra of the **1-EtOH**: standard (bottom) and dipolar-dephased (top). Recorded with a contact time of 4 ms. The dipolar filter was set to 50 μs.

Page 6: **Table S2.** Crystal data and structure refinement

Page 8: **Table S3**. Hydrogen bonds and stacking interactions geometry (in Å and °) in experimental and calculated structures

Page 11: **TableS4**. Aromatic analyzer score values for hierarchization of interactions between aromatic rings

Page 11: **Figure S5.** DSC curve for **1-MeOH**.

Page 12: **Table S5**. Interaction Energies (kJ/mol) calculated in Crystal Explorer program at B3LYP/6-31G(d,p) level of theory

Page 13: **Figure S6.** The crystal packing of: a) **Calc 1-H_2O**, b) **Calc 1-MeOH**, c) **Calc 1-EtOH**.

Table S1. ^{13}C NMR Chemical Shielding Constants (ppm) in experimental 1-MeOH and 1-EtOH (Exp), compared with those in calculated with and without solvates (Cal). The crystal structure of 1-MeOH and 1-EtOH determined by scXRD was used as a starting point for the geometry optimization. In the calculations, the positions of all atoms were optimized, while the cell parameters were fixed to their experimental values.

**Group**	**δ [ppm]**			**δ [ppm]**		
	Cal		Exp	Cal		Exp
	**1**[*]	**1-MeOH**	**1-MeOH**	**1**[#]	**1-EtOH**	**1-EtOH**
1-C	120.65	120.73	120.57	122.2	120.57	120.3
2-C	161.71	162.21	158.08	162.16	161.4	157.62
3-CH	112.53	112.42	112.74	113.22	112.59	113.17
4-CH	139.16	137.69	137.70	137.35	138.33	137.88
5-CH	122.98	123.00	123.37	120.75	124.93	122.00
6-C	133.58	132.54	131.78	132.08	132.05	131.76
7-C	167.85	168.38	168.39	167.31	167.38	168.19
8-CH_2	69.85	70.71	70.07	70.04	70.92	70.12
9-C	140.68	139.63	138.4	140.14	138.23	138.77
10-CH	132.6	131.13	128.66	131.57	130.44	130.62
11-C	140.68	140.23	141.84	140.68	141.06	138.77
12-CH	130.02	129.01	126.87	129.16	128.17	128.45
13-CH	128.91	131.60	128.66	130.5	133.36	131.76
14-CH	128.47	128.40	126.87	128.52	128.13	128.45
15-CH_2	61.45	62.06	62.43	61.55	62.47	62.35
16-CH_2	53.48	54.00	55.81	54.1	53.24	54.2
17-CH_2	47.11	47.26	49.57	47.51	47.82	49.62
18-CH_2	47.84	46.87	49.57	46.76	46.95	49.62
19-CH_2	48.47	48.10	52.24	48.58	48.29	49.62
20-C	140.47	141.21	141.84	142.06	142.24	142.08
21-C	155.86	155.23	152.79	155.58	155.23	153.03
22-CH	112.86	111.01	112.74	112.31	112.15	113.17
23-CH	123.71	122.44	123.37	122.48	122.35	122.00
24-CH	122.42	121.86	120.57	121.08	122.62	120.3
25-CH	119.66	119.03	119.55	119.37	119.39	119.8
26-CH_3	53.42	54.25	53.83	53.14	53.32	52.42
C1S	53.86	51.07	49.57		61.75	59.17
C2S					17.68	19.43

[*] MeOH was expelled from the experimentally obtained structure.

[#] EtOH was expelled from the experimentally obtained structure.

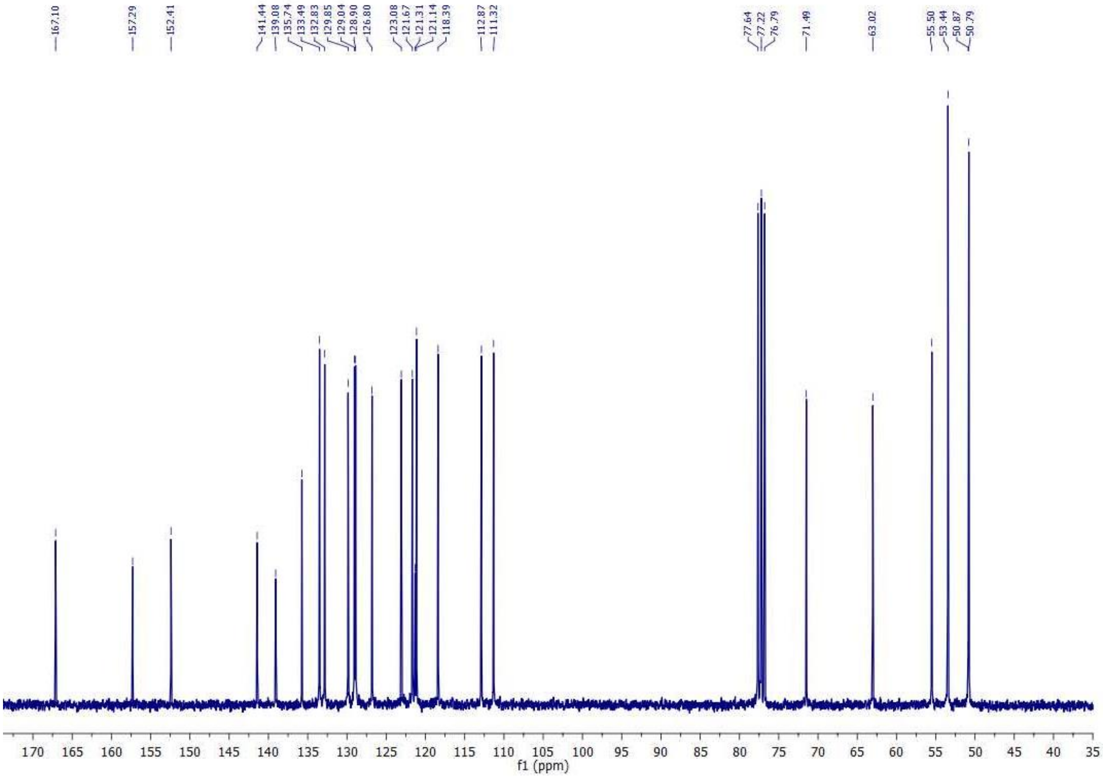

Figure S1. The ^{13}C NMR spectrum (in $CDCl_3$ to 300 MHz) of **1**.

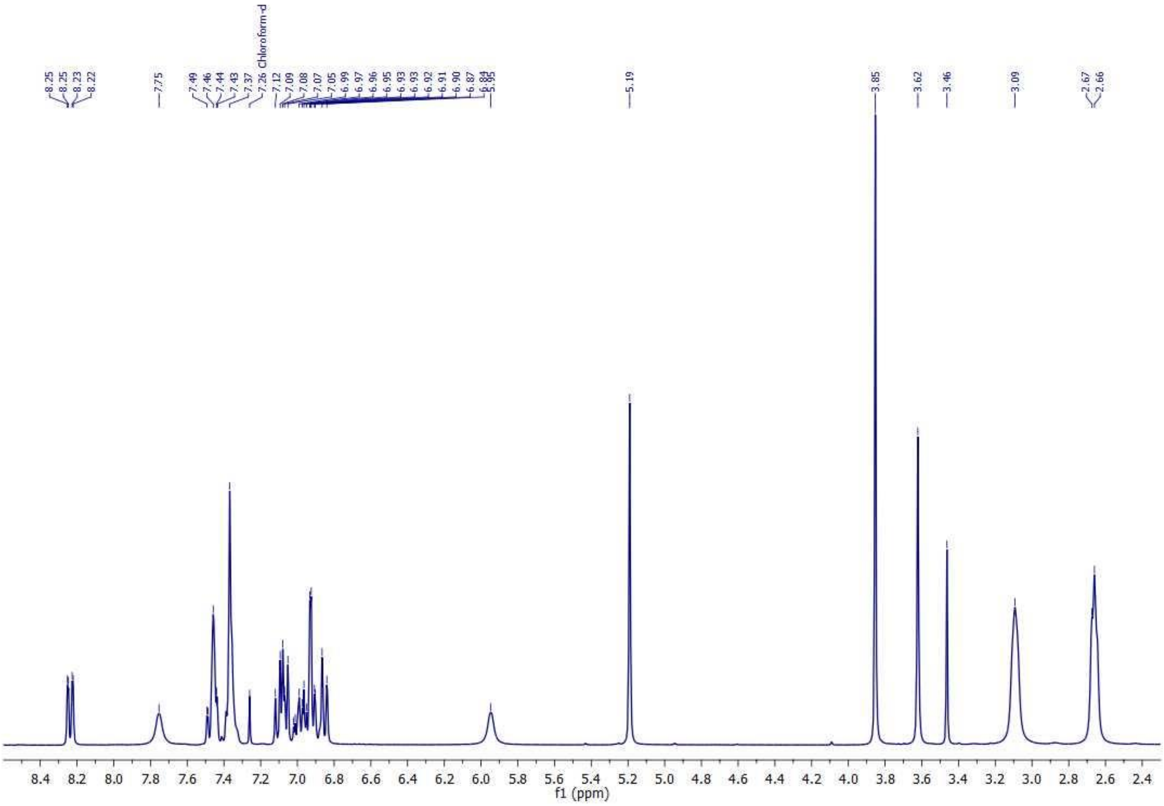

Figure S2. The ^{1}H NMR spectrum (in $CDCl_3$ to 300 MHz) of **1**.

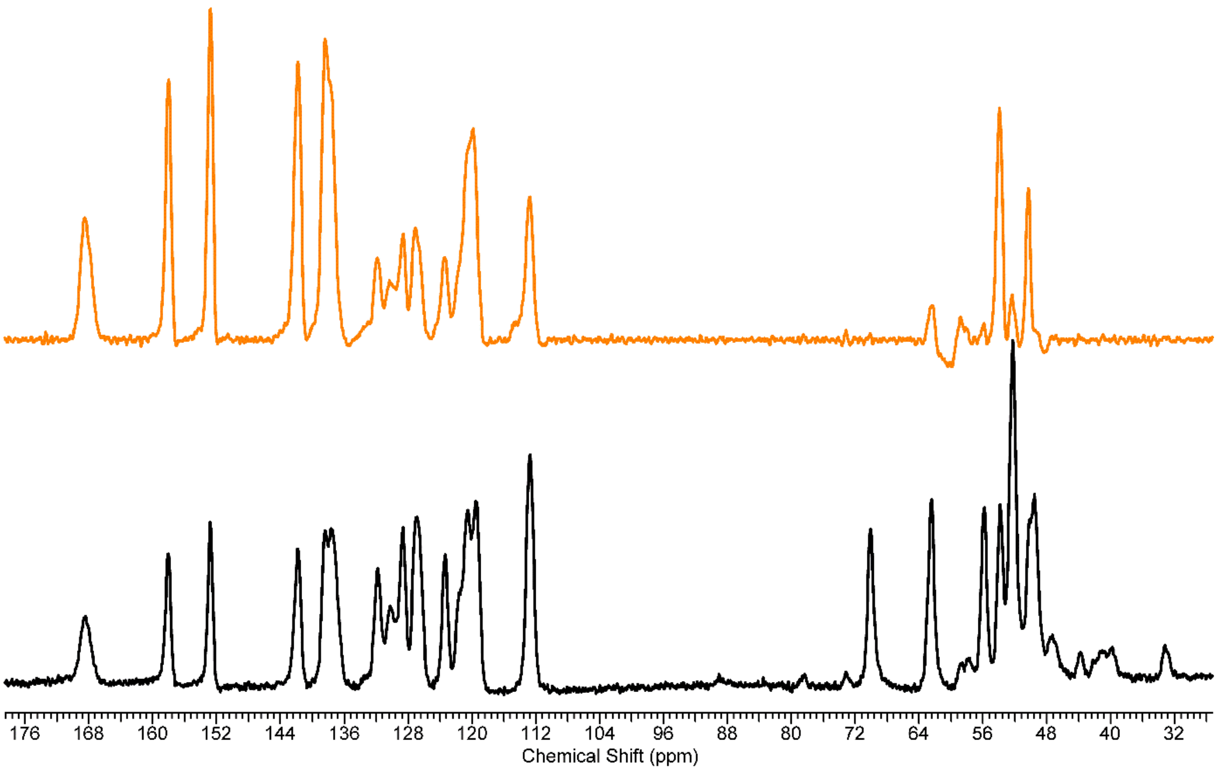

Figure S3. ^{13}C CP/MAS NMR spectra of the **1-MeOH**: standard (black) and dipolar-dephased (top). Recorded with a contact time of 4 ms. The dipolar filter was set to 50 μs.

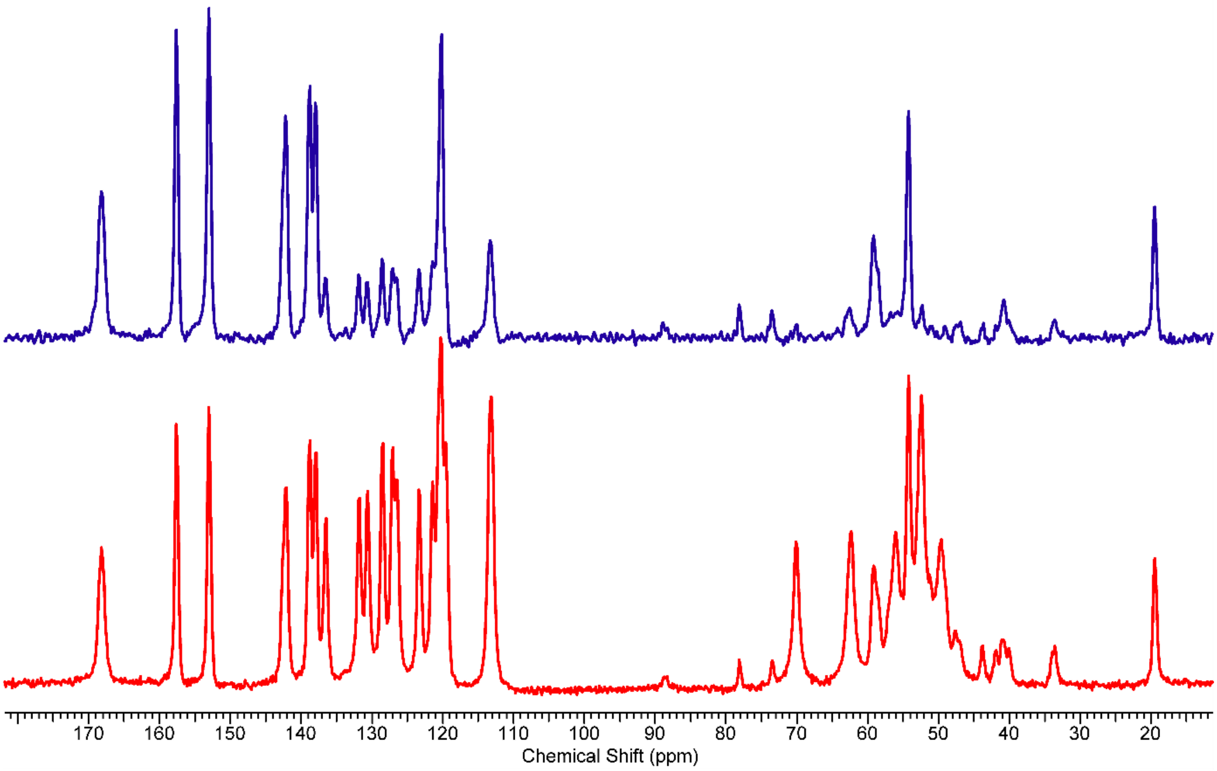

Figure S4. ^{13}C CP/MAS NMR spectra of the **1-EtOH**: standard (red) and dipolar-dephased (blue). Recorded with a contact time of 4 ms. The dipolar filter was set to 50 μs.

Table S2. Crystal data and structure refinement

Identification code	**1-MeOH**	**1-MeOH** (squeeze)	**1-MeOH_low**	**1-MeOH_low** (squeeze)
Empirical formula	$C_{26}H_{31}N_3O_3 \cdot 0.5$ CH_3OH	$C_{26}H_{29}N_3O_3$	$C_{26}H_{31}N_3O_3 \cdot 0.5$ CH_3OH	$C_{26}H_{29}N_3O_3$
Formula weight	447.54	431.52	447.54	431.52
Temperature/K	293.15		120	
Crystal system	*triclinic*			
Space group	*P*-1			
a/Å	7.2792(4)		7.2165(4)	7.2165(4)
b/Å	10.1555(5)		9.9585(6)	9.9585(6)
c/Å	17.1001(7)		17.0304(11)	17.0304(11)
α/°	76.527(4)		76.185(6)	76.185(6)
β/°	87.792(4)		87.712(5)	87.712(5)
γ/°	87.810(4)		87.901(5)	87.901(5)
Volume/Å3	1227.85(11)		1187.08(13)	1187.08(13)
Z	2		2	2
r_{calc} /g·cm^{-3}	1.211	1.167	1.252	1.207
μ/mm^{-1}	0.649	0.617	0.084	0.080
F(000)	478.0	460.0	478.0	460.0
Crystal size/mm^3	0.12 × 0.10 × 0.06		0.35 × 0.12 × 0.06	
Radiation/Å	Cu Kα (λ = 1.54184)		Mo Kα (λ = 0.71073)	
2*q* range for data collection/°	8.958 to 129.962		6.956 to 65.546	6.956 to 65.546
Index ranges	$-8 \le h \le 8$. $-11 \le k \le 11$. $-19 \le l \le 20$		$-10 \le h \le 10$. $-14 \le k \le 14$. $-25 \le l \le 25$	
Reflections collected	16651		38133	
Independent reflections	4107 [R_{int} = 0.0360]		8198 [R_{int} = 0.0606]	
Data/restraints/ parameters	4107/3/306	4107/2/298	8198/3/308	8198/2/298
Goodness-of-fit on F2	1.060	1.053	1.021	1.014
Final *R* indexes [*I*>=2σ (*I*)]	R_1 = 0.0562 wR_2 = 0.1704	R_1 = 0.0437 wR_2 = 0.1119	R_1 = 0.0697 wR_2 = 0.1576	R_1 = 0.0594 wR_2 = 0.1183
Final *R* indexes [all data]	R_1 = 0.0746 wR_2 = 0.1858	R_1 = 0.0606 wR_2 = 0.1223	R_1 = 0.1175 wR_2 = 0.1826	R_1 = 0.1032 wR_2 = 0.1353
Largest diff. peak/hole / e Å^{-3}	0.48/–0.61	0.14/–0.17	1.25/–0.55	0.35/–0.24

Table S2. Crystal data and structure refinement - continued

Identification code	**1-EtOH**	**1-EtOH** (squeeze)
Empirical formula	$C_{26}H_{31}N_3O_3 \cdot 0.5\ C_2H_6OH$	$C_{26}H_{29}N_3O_3$
Formula weight	454.55	431.52
Temperature/K	293.15	
Crystal system	*triclinic*	
Space group	*P*-1	
a/Å	7.5240(3)	
b/Å	10.1584(4)	
c/Å	16.9167(6)	
α/°	75.404(3)	
β/°	85.663(3)	
γ/°	85.721(4)	
Volume/Å3	1245.58(9)	
Z	2	
r_{calc} /g·cm^{-3}	1.212	1.151
μ/mm^{-1}	0.647	0.608
F(000)	486.0	460.0
Crystal size/mm^3	0.4 × 0.14 × 0.1	
Radiation/Å	CuKα (λ = 1.54184)	
2*q* range for data collection/°	9.01 to 133.778	
Index ranges	-8 ≤ h ≤ 8. -12 ≤ k ≤ 12. -20 ≤ l ≤ 20	
Reflections collected	26430	
Independent reflections	4401 [R_{int} = 0.0600]	
Data/restraints/ parameters	4401/2/304	4401/2/297
Goodness-of-fit on F2	1.059	1.049
Final *R* indexes [*I*>=2*σ* (*I*)]	R_1 = 0.0776; wR_2 = 0.2427	R_1 = 0.0398; wR_2 = 0.1077
Final *R* indexes [all data]	R_1 = 0.0946; wR_2 = 0.2634	R_1 = 0.0544; wR_2 = 0.1165
Largest diff. peak/hole / e Å^{-3}	0.77/-1.53	0.15/-0.13

Table S3. Hydrogen bonds and stacking interactions geometry (in Å and °) in experimental and calculated structures[*]

Structure	Interacting atoms	D-H	H…A	D…A	D-H…A
Interactions of the solvent molecule					
**calc 1-MeOH**	O1T-H1T…O1	0.98	2.75	3.441	128
	C1T-H1TB…O1	1.10	2.51	3.333	131
	O1T-H1T…O1T[i]	0.98	2.30	2.833	114
	C1T-H1TC_CgB[ii]	1.10	2.91	3.687	127
**calc 1-EtOH**	O1T-H1T….O1	0.98	1.90	2.840	160
interlayer	C1T-H1TB…O1T[i]	1.11	2.63	3.453	131
	C1T-H2TA…C9[iii]	1.10	2.64	3.634	151
	C5-H5…O1T[iv]	1.09	2.78	3.338	112
**calc 1-H_2O**	O1T-H1TA…O1	0.99	1.80	2.785	177
	O1T-H1TB…O1T[v]	0.97	2.47	3.092	122
Interactions in the $R^2_2(8)$ dimer (red motif)					
**1-MeOH**	N1-H1A…O1[vi]	0.87(2)	2.06(2)	2.925(3)	177(2)
**1-MeOH-low**		0.88(1)	2.02(1)	2.900(2)	175(1)
**1-EtOH**		0.86(1)	2.04(1)	2.901(2)	177(2)
**calc 1-MeOH**		1.04	1.77	2.805	178
**calc 1-EtOH**		1.04	1.80	2.835	178
**calc 1-H_2O**		1.03	1.81	2.848	177
**calc 1**		1.04	1.77	2.807	177
**1-MeOH**	N1-H1B…O2	0.90(2)	1.99(2)	2.682(2)	133(2)
**1-MeOH-low**		0.90(2)	2.00(1)	2.690(1)	133(1)
**1-EtOH**		0.86(1)	2.03(1)	2.685(2)	132(1)
**calc 1-MeOH**		1.02	1.92	2.6942	131
**calc 1-EtOH**		1.02	1.92	2.6900	130
**calc 1-H_2O**		1.02	1.90	2.6848	131
**calc 1**		1.02	1.93	2.7091	131
**1-MeOH**	C18-H18A…O3	0.97	2.45	3.010(2)	116
**1-MeOH-low**		0.99	2.43	3.000(2)	116
**1-EtOH**		0.97	2.48	3.0297(19)	115
**calc 1-MeOH**		1.10	2.32	2.9851	117
**calc 1-EtOH**		1.10	2.40	3.0334	115
**calc 1-H_2O**		1.10	2.36	3.0049	116
**calc 1**		1.10	2.33	2.9876	117
Interactions in the layer					
green motif					
**1-MeOH**	C3-H3…CgC[vii]	0.93	2.90	3.630(2)	136
**1-MeOH-low**		0.95	2.81	3.546(2)	135
**1-EtOH**		0.93	2.95	3.645(2)	133
**calc 1-MeOH**		1.09	2.72	3.5447	132
**calc 1-EtOH**		1.09	2.78	3.5808	130
**calc 1-H_2O**		1.09	2.64	3.480	133
**calc 1**		1.09	2.71	3.531	132
**1-MeOH**	C8-H8A…O3[vii]	0.97	2.76	3.407(2)	125
**1-MeOH-low**		0.99	2.71	3.353(2)	123
**1-EtOH**		0.97	2.73	3.416(2)	129

**calc 1-MeOH**		1.10	2.79	3.411	115
**calc 1-EtOH**		1.10	2.69	3.2689	112
**calc 1-H_2O**		1.10	2.75	3.401	117
**calc 1**		1.10	2.68	3.371	120
**1-MeOH**	C10-H10…O3^{vii}	0.93	2.75	3.471(2)	136
**1-MeOH-low**		0.95	2.63	3.377(2)	136
**1-EtOH**		0.93	2.96	3.681	135
**calc 1-MeOH**		1.09	2.51	3.3616	134
**calc 1-EtOH**		1.09	2.74	3.503	127
**calc 1-H_2O**		1.09	2.51	3.355	133
**calc 1-H_2O**		1.10	5.54	3.381	133
pink motif					
**1-MeOH**	C23-H23…C1^{viii}	0.93	3.03	3.675(2)	128
**1-MeOH-low**		0.95	2.94	3.606(2)	128
**1-EtOH**		0.93	3.04	3.691(2)	128
**calc 1-MeOH**		1.09	2.81	3.547	127
**calc 1-EtOH**		1.09	2.80	3.622	132
**calc 1-H_2O**		1.09	2.75	3.575	133
**calc 1**		1.09	2.76	3.548	129
**1-MeOH**	C26-H26A…CgB^{viii}	0.96	2.94	3.562(2)	123
**1-MeOH-low**		0.98	2.81	3.475(2)	125
**1-EtOH**		0.96	2.90	3.558(2)	126
**calc 1-MeOH**		1.10	2.90	3.524	116
**calc 1-EtOH**		1.10	2.69	3.506	130
**calc 1-H_2O**		1.10	2.65	3.434	128
**calc 1**		1.10	2.60	3.436	126
**1-MeOH**	C22-H22…O2 ^{viii}	0.93	2.71	3.589(2)	157
**1-MeOH-low**		0.95	2.64	3.525(2)	155
**1-EtOH**		0.93	2.74	3.619(2)	157
**calc 1-MeOH**		1.09	2.54	3.566	156
**calc 1-EtOH**		1.09	2.46	3.444	150
**calc 1-H_2O**		1.09	2.57	3.559	151
**calc 1**		1.09	2.49	3.497	153
yellow motif					
**1-MeOH**	C7…C2^{ii}			3.499(3)	
**1-MeOH-low**				3.381(2)	
**1-EtOH**				3.476(2)	
**calc 1-MeOH**				3.511	
**calc 1-EtOH**				3.394	
**calc 1-H_2O**				3.317	
**calc 1**				3.323	
**1-MeOH**	C14-H14…C6^{ii}	0.93	2.88	3.762(2)	158
**1-MeOH-low**		0.95	2.80	3.700(2)	160
**1-EtOH**		0.93	2.98	3.852(2)	157
**calc 1-MeOH**	assisted by interactions with MeOH	1.09	2.71	3.691	149
**calc 1-EtOH**		1.09	2.86	3.866	153
**calc 1-H_2O**		1.09	2.59	3.616	157
**calc 1**		1.09	2.59	3.612	157
other interactions					

**1-MeOH**	C17-H17A…C22[ix]	0.97	3.04	3.493(2)	110
**1-MeOH-low**		0.99	2.94	3.418(2)	111
**1-EtOH**		0.97	2.98	3.474(2)	113
**calc 1-MeOH**		1.11	2.89	3.399	108
**calc 1-EtOH**		1.11	2.65	3.332	119
**calc 1-H_2O**		1.11	2.90	3.411	108
**calc 1**		1.11	2.84	3.400	111
Interlayer interactions					
blue motif					
**1-MeOH**	C25-H25…C12[x]	0.93	3.19	3.974(2)	143
**1-MeOH-low**		0.95	3.10	3.917(2)	145
**1-EtOH**		0.93	3.27	4.053(2)	143
**calc 1-MeOH**		1.09	3.12	4.013	140
**calc 1-H_2O**		1.09	3.00	3.938	145
**calc 1**		1.09	2.96	3.888	144
**1-MeOH**	C19-H19A…N2[x]	0.97	2.88	3.713(2)	144
**1-MeOH-low**		0.99	2.78	3.621(2)	143
**1-EtOH**		0.97	2.90	3.713(2)	142
**calc 1-MeOH**		1.11	2.73	3.707	146
**calc 1-H_2O**		1.11	2.57	3.557	148
**calc 1**		1.11	2.62	3.604	148
other interactions					
**1-MeOH**	C13-H13…C5[xi]	0.93	3.23	3.793	121
**1-MeOH-low**		0.95	3.23	3.744	116
**1-EtOH**		0.93	3.18	3.714	118
**calc 1-MeOH**		1.09	3.04	3.618	114
**calc 1-EtOH**		1.09	2.95	3.585	118
**calc 1-H_2O**		1.09	3.06	3.557	108
**calc 1**		1.09	3.08	3.644	113
**1-MeOH**	C12-H12…C23[xii]	0.93	3.21	3.901	133
**1-MeOH-low**		0.95	3.09	3.784	131
**1-EtOH**		0.93	3.26	3.965	135
**calc 1-MeOH**		1.09	2.95	3.759	132
**calc 1-H_2O**		1.09	2.87	3.660	129
**calc 1**		1.09	2.97	3.749	129

[*] Calculated structures with optimized all atoms and unit cell parameters. The parameters are given without standard deviations. Cg denotes the molecular center of gravity.

[i] 2-x,-y,-z; [ii] 1-x,1-x,-z; [iii] 1+x,1+y,z; [iv] 1-x,-y,-z; [v] 2-x,-y,1-z; [vi] 2-x,1-y,-z; [vii] 1-x,1-y,1-z; [viii] 2-x,1-y,1-z; [ix] x-1,y,z; [x] 1-x,2-y,1-z; [xi] x,1+y,z ; [xii] 2-x,2-y,1-z,

Table S4. Aromatic analyzer score values for hierarchization of interactions between aromatic rings*

Interaction**	**1-MeOH**	**1-MeOH-low**	**1-EtOH**	**calc 1-MeOH**	**calc 1-EtOH**	**calc 1-H_2O**	**calc 1**
C3-H3…CgC^{vii}	9.2	9.3	9.2	9.2	9.2	8.8	9.2
C23-H23…C1^{viii}	7.5	7.5	7.4	7.5	7.1	7.2	7.2
C7…C2^{ii}	6.1	6.6	6.9	6.1	7.7	6.8	7.1
C12-H12…C23^{xii}	5.9	6.3	5.7	6.1		6.2	6.3
C13-H13…C5^{xi}	5.3	5.5	5.4	5.9	5.8	6.2	5.9
C14-H14…C6^{ii}	5.4	5.3	5.3	5.6	5.1	4.9	5.0
C25-H25…C12^{x}	4.7	5.0	4.7	4.3		5.0	5.1

[*]The interactions assessed as strong (score 7-10) are marked in red while the medium ones (score 3-7) are in black and limited to those above 4.0.
[**] Symmetry codes and colors correspond to the motives found in crystals (see Table S3 and Figure 7).

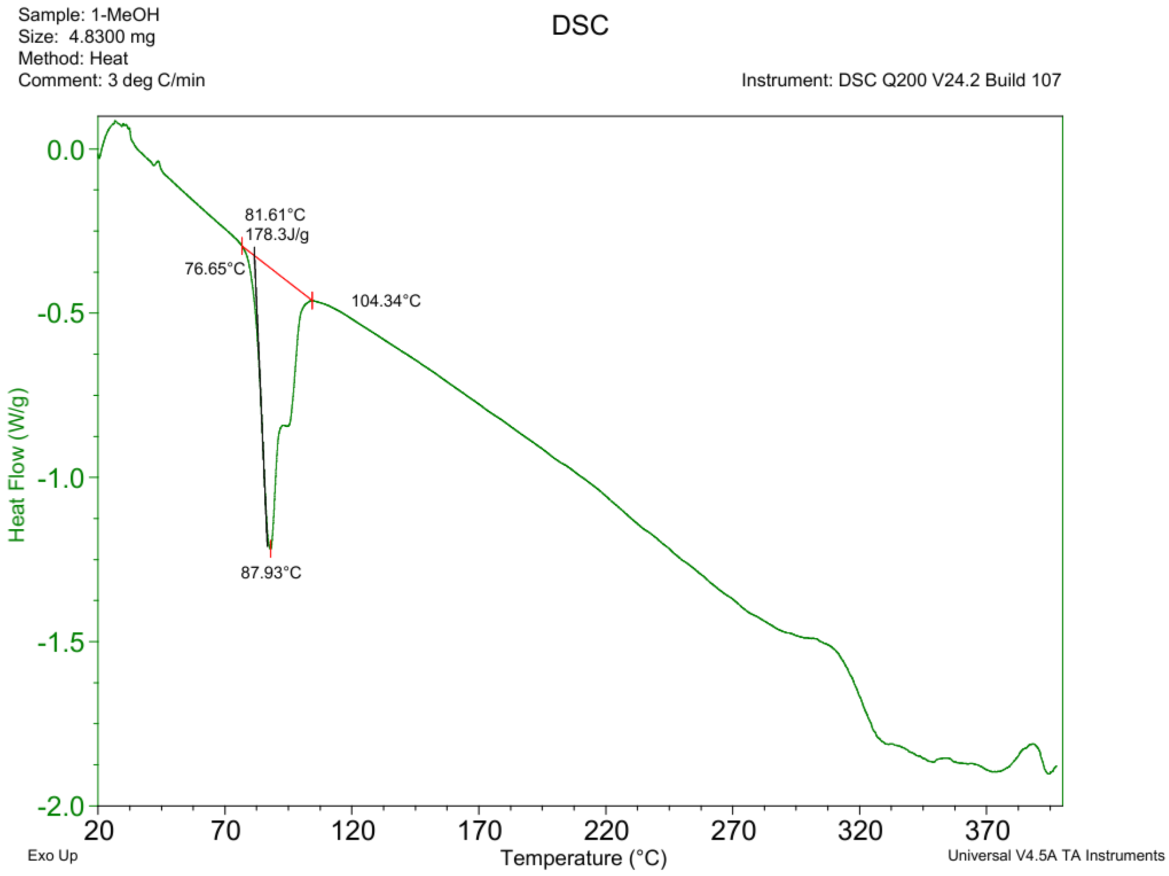

Figure S5. DSC curve for **1-MeOH**.

Table S5. Interaction Energies (kJ/mol) calculated in Crystal Explorer program at B3LYP/6-31G(d,p) level of theory

Symmetry operation	R*	E_ele	E_pol	E_dis	E_rep	E_tot**
**1-MeOH**						
1-x,1-y,1-z	5.04	-31.8	-8.5	-104.8	66.3	-90.2
2-x,1-y,-z	14.00	-71.8	-18.1	-12.5	67.5	-58.5
2-x,1-y,1-z	6.72	-11.6	-3.2	-82.3	50.4	-55.1
1-x,1-x,-z	13.63	-17.8	-6.3	-54.6	30.0	-52.5
1-x,2-y,1-z	10.14	-7.2	-1.1	-49.8	28.0	-34.6
x-1, y, z	7.28	-1.5	-2.6	-34.9	13.4	-25.6
2-x,2-y,1-z	11.33	-1.6	-0.6	-29.5	10.7	-21.2
**1-MeOH-low**						
1-x,1-y,1-z	4.96	-38.7	-9.8	-115.9	86.3	-95.8
2-x,1-y,-z	13.96	-75.5	-19.0	-12.8	74.4	-59.1
2-x,1-y,1-z	6.68	-15.2	-3.8	-89.2	62.4	-58.0
1-x,1-x,-z	13.59	-22.7	-7.1	-62.3	42.2	-57.4
1-x,2-y,1-z	10.01	-9.8	-1.5	-55.7	36.7	-37.3
x-1, y, z	7.22	-2.8	-3.0	-38.5	17.8	-27.7
2-x,2-y,1-z	11.20	-3.1	-0.8	-33.4	16.2	-23.0
**1-EtOH**						
1-x,1-y,1-z	4.99	-30.5	-7.9	-101.2	62.8	-87.5
2-x,1-y,-z	13.84	-76.5	-19.4	-13.2	75.6	-60.1
2-x,1-y,1-z	6.73	-11.5	-3.3	-82.0	48.0	-56.4
1-x,1-x,-z	13.79	-17.4	-6.0	-56.6	30.5	-53.3
1-x,2-y,1-z	9.98	-6.7	-1.1	-49.9	27.0	-34.6
x-1, y, z	7.52	-1.5	-2.2	-30.8	11.2	-23.2
2-x,2-y,1-z	11.47	-1.3	-0.6	-28.1	9.1	-20.6

[*] R is the distance between molecular centroids (mean atomic position) in Å.
[**]Total energies are the sum of the four energy components, scaled as follows:
E_tot = 1.057×E_ele + 0.740×E_pol + 0.871×E_dis + 0.618×E_rep

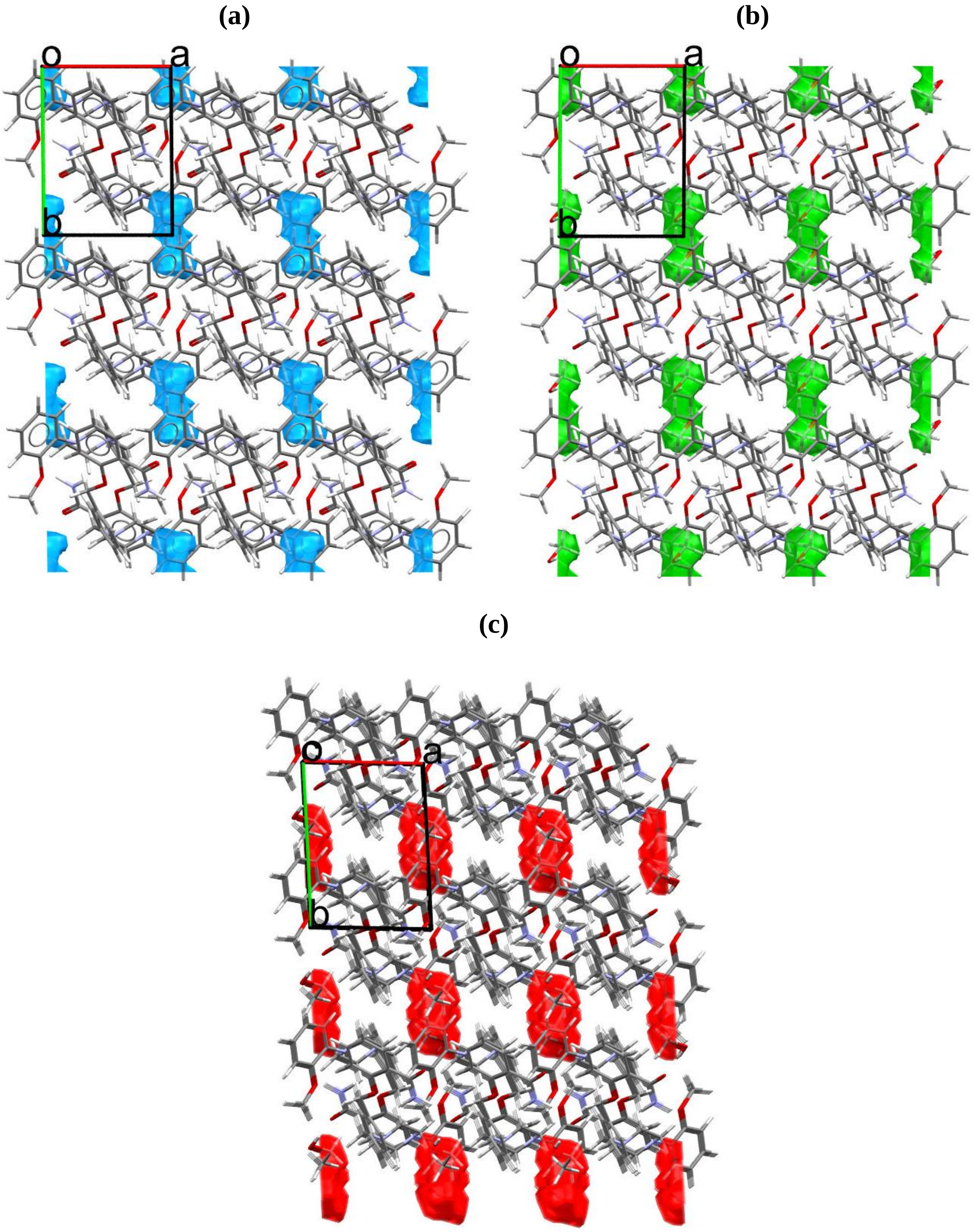

Figure S6. The crystal packing of: (a) **Calc 1-H_2O**, (b) **Calc 1-MeOH**, (c) **Calc 1-EtOH**. The space occupied by solvent was calculated in Mercury 2020.2.0 program.
